# Innovations in Deaf Health Care Communication: Systematic Review of Sign Language Recognition Systems

**DOI:** 10.2196/70417

**Published:** 2026-04-09

**Authors:** Milena Soriano Marcolino, Lucca Fagundes Ramos de Oliveira, Lucas Rocha Valle, Luiza Marinho Motta Santa Rosa, Gabriela Teodora de Souza Sanches, Natalia Sales Santos, Michelle Ralil da Costa, Elidea Lucia Almeida Bernardino, Raniere Alislan Almeida Cordeiro, Raquel Oliveira Prates, Zilma Silveira Nogueira Reis, Mario Fernando Montenegro Campos

**Affiliations:** 1 Medical School, Universidade Federal de Minas Gerais, Belo Horizonte, Brazil; 2 Telehealth Center, University Hospital, Universidade Federal de Minas Gerais, Avenida Professor Alfredo Balena, 190, Santa Efigênia, Belo Horizonte, CEP 30130-100, Brazil, +55 31 3307 9201; 3 Institute for Health Technology Assessment, Porto Alegre, Brazil; 4 Medical School, Universidade Federal de Ouro Preto, Ouro Preto, Brazil; 5 Faculdade de Ciências Médicas de Minas Gerais, Belo Horizonte, Brazil; 6 Department of Computer Science, Universidade Federal de Minas Gerais, Belo Horizonte, Brazil; 7 Faculty of Arts and Sciences, Universidade Federal de Minas Gerais, Belo Horizonte, Brazil

**Keywords:** computer neural network, artificial intelligence, biomedical technology, sign language, hearing loss, deafness, communication barriers, gestures, PRISMA, Preferred Reporting Items for Systematic Reviews and Meta-Analyses

## Abstract

**Background:**

Deaf individuals often face communication challenges when interacting with those who can hear. Within health care settings, these challenges may pose risks to their safety, potentially resulting in misdiagnoses, treatment errors, and decreased quality of care.

**Objective:**

This study aims to systematically review the evidence on communication systems reported in the literature that use human-computer interaction techniques to support communication between deaf individuals who use sign language and hearing health professionals in health care settings. The review focuses on systems that are either currently in use or proposed for use in health care and that have been tested using human participants or videos of human users.

**Methods:**

A comprehensive search was performed via MEDLINE, Web of Science, ACM, IEEE Xplore, Scopus, and Google Scholar in March 2025. The inclusion criteria comprised studies developing a sign language recognition system within a health care context and testing with human users. Eligible studies underwent screening by 2 independent investigators (LRV and LMMSR or LFRdO and GTdSS), with any disagreements resolved by a senior researcher (MSM).

**Results:**

The search retrieved 21,778 publications, and screening of reference lists identified 2 additional studies, resulting in a total of 23 studies meeting the eligibility criteria. Most systems (15/23, 65.2%) were image-based, while 34.8% (8/23) relied on sensors (glove-based or depth-sensing). Applications varied across health care settings, including general hospital care (10/23, 43.5%), emergencies (8/23, 34.8%), and primary care (4/23, 17.4%). All systems were in the development and testing stage, with no data on security and psychological impacts. Accuracy ranged from 25% to 100% for image-based and 72% to 99.7% for sensor-based systems. Bidirectionality and facial expression recognition, crucial for effective communication, were largely overlooked.

**Conclusions:**

Image-based systems were more common than sensor-based ones, though both showed wide variability in accuracy in recognizing and interpreting signs. Most systems failed to address critical aspects such as bidirectional communication and the recognition of facial expressions, essential for effective communication. None fully addresses the requirements for integration into health care settings. These findings highlight the need for further research on implementation, usability, and impact on the quality of care for deaf patients.

## Introduction

Sign languages are a form of communication characterized by the successful coordination of gestures, body, head, and hand motions, with facial expressions. They are naturally developed and highly structured systems, governed by a set of linguistic rules, different from spoken languages with no standardized written forms [1]. They enable comprehensive and rich linguistic systems for formulating words and phrases, making them the primary mode of communication for many hearing-impaired people [2]. According to the World Health Organization, over 430 million people worldwide experience disabling hearing loss, a number expected to exceed 700 million by 2050 [3]. Despite the World Federation of the Deaf estimating that there are over 200 sign languages and over 70 million deaf individuals who use them, reliable data on sign language users remains scarce [4].

Even though sign language is the primary mode of communication for millions of deaf individuals, they still encounter significant barriers in daily interactions, particularly in critical areas such as health care, education, and public services, where most professionals do not understand or use sign language [5-7]. Specifically in health care, this communication gap can lead to misdiagnoses, inadequate treatment, and reduced quality of care [8].

In response to this challenge, and recognizing the significance of sign language in fostering social integration for the deaf community, researchers have developed sign language recognition (SLR) systems. SLR refers to the identification and interpretation of sign language gestures and movements [9]. These systems aim to facilitate real-time translation between sign and spoken languages, to build a communication bridge between deaf individuals who communicate through sign language and ordinary people, and to enhance accessibility and inclusion across multiple domains, including health care [10]. In this pursuit, machine learning and other artificial intelligence (AI) techniques have become important emerging tools to help overcome communication barriers, enhancing not only accessibility but also valuing the cultural identity of the deaf community.

Regarding automatic SLR systems, it is crucial to highlight the use of advanced algorithms and other AI techniques to interpret the complex gestures and movements inherent to sign language communication. With extensive use of advanced computer vision algorithms and machine learning techniques, these systems are becoming increasingly able to automatically recognize and translate signs into text of the target language, thereby facilitating effective communication between deaf individuals and those who do not understand sign language [11-13].

The SLR systems comprise different types of methodologies and technologies to recognize and translate sign language. In this paper, we organize them into 2 main categories: image-based and sensor-based. Even though strictly speaking, cameras are sensors, we used sensor-based to identify the approaches that use other sensor modalities. Image-based approaches use computer vision techniques coupled with deep learning models to analyze video streams from cameras that capture hand gestures, body movements, and facial expressions to accurately detect and interpret signs [11,13,14]. Sensor-based systems use wearable devices equipped with sensors, such as accelerometers and gyroscopes, to capture hand and body movements and translate signs into written or spoken language [11,12,15]. These systems can be divided into 2 subgroups: glove-based systems and depth-sensor systems. Glove-based systems use specialized gloves embedded with sensors to capture fine-grained hand movements, enabling real-time translation into spoken or written language. In contrast, depth-sensor systems rely on depth-sensing cameras, such as Kinect or light detection and ranging, to obtain 3D information about hand and body movements. By analyzing the 3D pose of different body parts, these systems allow more precise recognition and interpretation of sign language gestures [16].

Each of the aforementioned types of systems has strengths and limitations concerning accuracy in detecting and interpreting signs, portability, cost, and accessibility. Additionally, user-friendliness is an important consideration in the development and implementation of SLR systems. It is critical for ensuring accessibility and inclusion for deaf individuals. As these systems are intended to be used by people with varying levels of technical expertise, their design must prioritize simplicity, intuitiveness, and ease of use [17]. This is particularly true in health care settings, where effective communication with medical staff is essential. Furthermore, given the sensitive nature of health care interactions, it is crucial to assess how these systems address ethical concerns, such as patient data privacy, and practical challenges related to accessibility, including cost and implementation feasibility in diverse health care settings.

Therefore, this study aims to systematically review the evidence on the translation systems developed for deaf people who communicate through sign language with hearing health professionals in a health care context, which are already in use or proposed for use and have been tested with human users or videos of human users. The main research question was: What technologies have been developed and tested in real-world settings to translate sign and oral languages, facilitating communication between deaf patients who primarily use sign language and health care workers? The specific questions are as follows:

- In which context of health care have these technologies been used?
- Which languages (sign and oral) can these technologies translate?
- Which technologies are required for it to be used on-site? How were they developed?
- How were they deployed and tested?
- How has the communication between health care workers and deaf people been improved by using these technologies?
- How was the efficacy of these technologies evaluated?
- Is the system or technology “bidirectionally” interactive?
- How do these systems address ethical concerns in health care settings, such as patient privacy and data security?

## Methods

### Overview

The research protocol was registered in the Open Science Framework and previously published in detail [18,19]. It followed guidance from the Cochrane Guidelines and the PRISMA (Preferred Reporting Items for Systematic Reviews and Meta-Analyses) statement [20,21] (Checklist 1). A multidisciplinary team comprising researchers from health and computing domains, along with linguistic specialists in sign language, collaboratively conducted the systematic review. Two members of the team are linguistic specialists and sign language researchers, one of them is deaf.

### Search Strategy

Independent researchers performed a literature search using Web of Science, MEDLINE, IEEE Xplore, ACM, Scopus, and Google Scholar. A preliminary search strategy, developed by 5 authors (MSM, LFRO, LRV, ROP, and ZSNR), incorporated MeSH and defined text words relevant to the topic (Multimedia Appendix 1). The last search was conducted on March 27, 2025.

All studies, regardless of publication date or language, from inception onward were considered. In the case of unpublished studies, the authors were contacted at least 3 times via email or other social networks used by researchers to request additional information. Reference lists of eligible studies were examined to identify additional eligible studies.

### Study Selection

Prospective, retrospective, or descriptive studies that address the development of communication systems specifically designed for deaf individuals in health care encounters and that involve testing with human users or videos of human users were included. Human users could be people of any age who are deaf and whose primary communication modality is sign language.

Studies that do not address the specified research questions, do not mention testing with human users or videos of human users, or do not mention use in health care encounter contexts were excluded. Short communications, conference abstracts, and correspondences were not excluded.

Independent researchers blindly screened the studies. Titles and abstracts of identified studies were individually reviewed to assess eligibility. Full-text versions of papers that were not excluded at this initial stage were read for a thorough examination. Subsequently, potentially pertinent studies were independently evaluated to ascertain if they aligned with the inclusion criteria. Any disagreements were resolved by a senior researcher (MSM). Whenever necessary, corresponding authors were contacted to obtain data not included in the publication using email and ResearchGate.

Search data for the identified studies and information for each stage of study selection were registered in detail, following the guidelines of the PRISMA methodology [20,21].

### Data Extraction

A data extraction table was custom-designed for this study and independently piloted by 2 researchers, as well as data extraction. The extraction was checked by 2 other reviewers, one researcher with a computer science background and a senior researcher with a health science background. Conflicts were resolved by consensus or by consulting a senior researcher (MSM). Details on the variables extracted were previously published [18,19].

Furthermore, the researchers responsible for each study included in the systematic review were contacted via email (with up to 3 attempts) and ResearchGate (at least 1 attempt) to obtain updates on the current status of their systems and to request any additional information regarding their application in real-world contexts.

The definitions used regarding the type of SLR system, the corpus formation, and the health context are available in Multimedia Appendix 2. To extract data from papers that met the eligibility criteria, the authors of the study developed a codebook with clear definitions for all variables to ensure consistent data collection (Multimedia Appendix 3).

### Data Analysis

A qualitative synthesis was performed to analyze the data, and a narrative synthesis of the evidence was conducted to provide an overview of the results. The results are summarized according to system types: image-based and sensor-based.

## Results

### Search Results and Study Selection

The search retrieved 21,778 publications, 2021 of which were duplicates. Most studies were excluded after title and abstract analysis (n=19,605). In total, 6 reports were unavailable, of which 4 did not respond despite repetitive contact attempts with corresponding authors, and 2 did not make any contact details available (Multimedia Appendix 4), thus leaving 146 selected for full-text screening. Of those, 22 studies were selected for inclusion after applying the eligibility criteria. In 2 cases, publications by the same research groups were identified. The first case involved a deep learning–based system for recognizing emergency gestures in Indian Sign Language to support communication with hearing-impaired individuals [22,23]. The journal paper published in IEEE Access in 2022 represents an extended and more comprehensive version of the earlier conference paper. It includes additional models (such as 3D convolutional neural network [CNN] and You Only Look Once [YOLO] v5), a more detailed methodology, a larger dataset, and more robust performance metrics, including mean average precision, precision, and recall. Given its methodological completeness and broader evaluation, only the IEEE Access paper was included in this review, while the conference version was excluded to avoid duplication of data and analysis [22].

The second case involved 2 publications from a Brazilian research group describing a system for recognizing Brazilian Sign Language (Libras) in the health context [15,24]. The 2024 journal paper incorporates a more advanced architecture (multiple-stream versus 2-stream), enhanced performance results, and a more detailed methodology, while using the same dataset as the earlier work. Thus, only the 2024 version was included [15].

**Figure 1.**
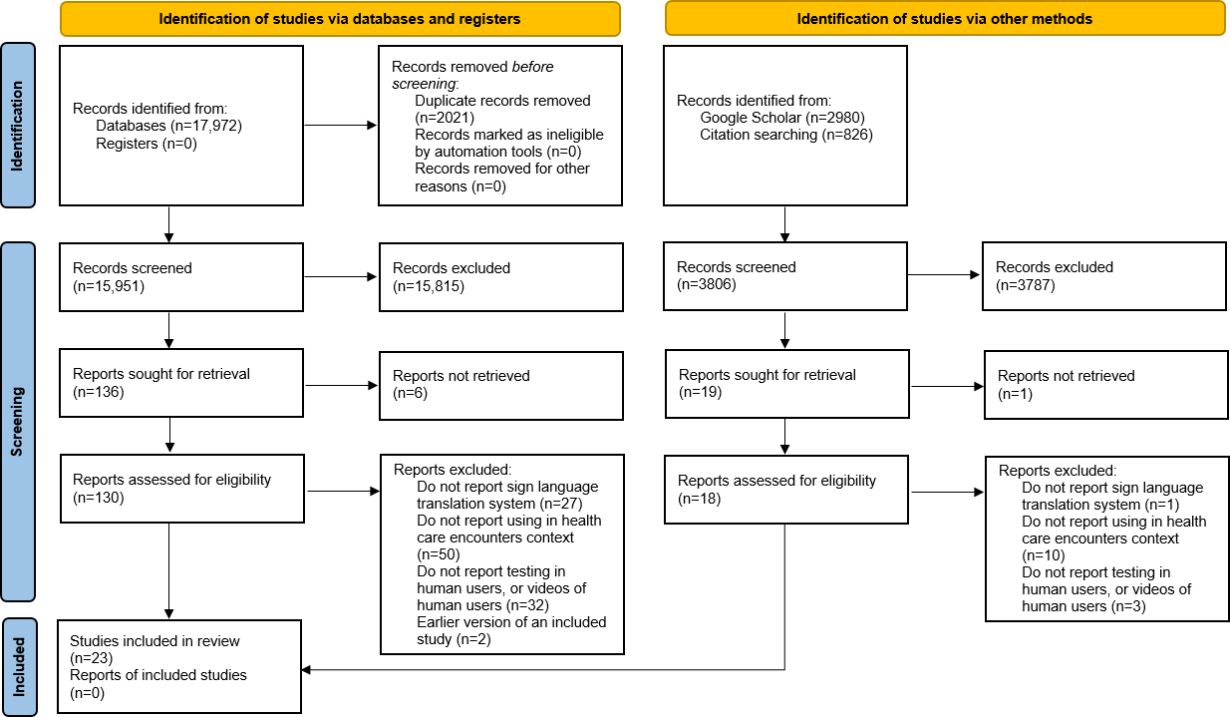
PRISMA (Preferred Reporting Items for Systematic Reviews and Meta-Analyses) flowchart [21].

Additionally, 2 studies were obtained from reference list screening, totaling 23 studies ultimately included in the review (Figure 1) [12-15,22,25-42].

### Characteristics of Included Studies

The main characteristics of the included studies are summarized in Tables1  2 and Multimedia Appendix 5 [12-15,22,25-42]. They were published in 18 journals and conferences from 2015 to 2024. All of them were published in English. In 15 studies, the system was classified as image-based [13-15,22,25-28,30,31,36-39,42], and in 7, it was sensor-based. Of these, 4 were depth-sensing [29,33,35,41], and 3 were glove-based [32,34,40].

**Table 1.** Main architectural, functional, and technical characteristics of sign language recognition systems.

	Languages involved (oral and sign)	Technology needed		Infrastructure needed
		Hardware or software	Development technology^a^	
Image-based				
Xia et al (2022) [13]	Chinese and Chinese Sign Language	Kendryte K210, 8-megapixel camera, microphone arrays, operating system FreeRTOS, LabelImg, Autodesk Inventor	TensorFlow, Keras, Darknet	SLA^b^ 3D printing technology and heart-speaker device
Pikoulis et al (2022) [14]	Greek and Greek Sign Language	No information	Sentence-BERT^c^, Google Mediapipe	Computer and camera
da Silva et al (2024) [15]	Brazilian Portuguese and Brazilian Sign Language (Libras)	Intel Core i3 and all experiments were performed on Ubuntu 18.04 LTS^d^	LSTM^e^ network, OpenPose, Keras, cuDNN^f^ 8.5	Computer and smartphone HD^g^ camera
Das et al (2023) [25]	English and Indian Sign Language	No information	CNN^h^, BiLSTM^i^	Computer and camera
Ko et al (2019) [26]	Korean and Korean Sign Language	No information	OpenPose, PyTorch	Computer and camera
Barrientos-Villalta et al (2022) [27]	Peruvian Spanish and Peruvian Sign Language	Google Cloud, Storage, Google Cloud Run	Google Mediapipe, LSTM neural network	Mobile devices and internet
Ramírez Sánchez et al (2021) [28]	Spanish and Mexican Sign Language	Webcam	Google MediaPipe, OpenCV, CNN, HMM^j^, Viterbi algorithm	Webcam and computer
Gandhi et al (2021) [30]	English and Indian Sign Language	Mobile phones with their camera resolutions	CNN using Image Stacking (VGG-16^k^ and Resnet50) CNN + LSTM and LSTM with OpenPose	Mobile phone and camera
Uchil et al (2019) [31]	English and Indian Sign Language	Intel Core i5 CPU^l^ running macOS Mojave and Core i3 CPU running Windows 7, smartphone	OpenPose library, OpenCV	Mobile phone camera
Areeb et al (2022) [22]	English and Indian Sign Language	No information	3D CNN, pretrained VGG-16, LSTM (RNN-LSTM)^m^ scheme, YOLO^n^ v5	Computer and camera
Adithya and Rajesh (2020) [36]	English and Indian Sign Language	Digital camera	LSTM network	Computer and camera
Ihsan et al (2024) [37]	English and American Sign Language	Intel Core i5, Visual Studio Code	Pretrained CNN (MobileNetV2) model, BiLSTM model, Mediapipe, TensorFlow, Keras	Computer, camera, and smartphones
Das et al (2024) [38]	English and Indian Sign Language	No information	CNNs, LSTM, long-term recurrent convolutional network model	Computer and mobile phone camera
Faisal et al (2023) [39]	Arabic and Saudi Sign Language	Robot Operating System, IClone Pro, 3DXchange, Unity	Self-developed concise 3D graph convolutional network, time delay neural network model, DTW^o^ algorithm, FastSpeech2, Kaldi toolkit, MediaPipe	Portable electronic devices or computers equipped with a camera
Bellil et al (2024) [42]	Arabic and Algerian Sign Language	iPhone XS MAX	1D-CNN, MediaPipe	Computer and smartphone
Sensor-based (depth-sensing)				
Hisham and Hamouda (2019) [29]	Egyptian Arabic and Egyptian Arabic Sign Language	Kinect SDK, Windows 7, Visual Basic.NET, C# and C++	Bayesian network, Ada-Boosting, DTW, and HMM	Computer and Microsoft Kinect sensor
Sarhan et al (2015) [33]	Arabic and Arabic Sign Language	No information	HMM	Computer and Microsoft Kinect sensor
Süzgün et al (2015) [35]	English and Turkish Sign Language	Personal computer and a touch display	DTW algorithm	Computer and Microsoft Kinect sensor
Dewasurendra et al (2020) [41]	Sinhala and Sri Lankan Sign Language	Webcam, mobile device with camera, microphone, GPS	EfficientNet-Lite0, Pillow (Python image processing library), MaryTTS Framework, CMU^p^ Sphinx 4 toolkit (Sphinx4+ SphinxTrain) and natural language processing, TensorFlow	Kinect and Leap Motion Controller
Sensor-based (glove-based)				
Deji Dere et al (2022) [32]	English and American Sign Language	Arduino Nano 33 BLE, Edge Impulse software	1D-CNN model	Inertial Measurement Unit
Guo et al (2023) [34]	English and American Sign Language	INMO AIR with Android 10 Go	Android-MediaPipe, VOSK^q^ library	Mobile phone and augmented reality glasses^r^
Luqman and Mahmoud (2020) [40]	Arabic and Arabic Sign Language	No information	KenLM (statistical language model), MADAMIRA^s^ (morphological analyzer and disambiguation tool)	Computer and camera
Hybrid				
Sosa-Jiménez et al (2022) [12]	Mexican Spanish and Mexican Sign Language	Intel Core i7 running Windows 7, Intel Core i5 running Windows 8.1, Microsoft Visual Studio 2015	Markov models (probabilistic) and neural networks	Computer and Microsoft Kinect sensor

a Artificial intelligence and imaging processing.

b SLA: stereolithography.

c BERT: bidirectional encoder representations from transformers.

d LTS: long-term support.

e LSTM: long short-term memory.

f cuDNN: CUDA deep neural network library.

g HD: high definition.

h CNN: convolutional neural network.

i BiLSTM: bidirectional long short-term memory.

j HMM: hidden Markov model.

k VGG-16: visual geometry group 16 layers.

l CPU: central processing unit.

m RNN-LSTM: recurrent neural network with a long short-term memory.

n YOLO: You Only Look Once.

o DTW: dynamic time warping.

p CMU: speech recognition toolkit.

q VOSK: offline speech recognition toolkit [43].

r Mobile phones and augmented reality glasses: portable electronic devices that enable interactive digital experiences.

s MADAMIRA: a system for morphological analysis and disambiguation of Arabic [44].

**Table 2.** Main characteristics of the recognition approaches of sign language recognition systems.

	Corpus formation			Captures facial expressions and body movement	Health context
	Type	Isolated words	Sentences		
Image-based					
Xia et al (2022) [13]	Isolated words	19	0	N/A^a^	General hospital care
Pikoulis et al (2022) [14]	Isolated words and sentences	6319 words with 1374 of them being unique (excluding repetitions)	1029 simple sentences with 945 of them being unique (excluding repetitions).	N/A	Psychiatric interviews
da Silva et al (2024) [15]	Isolated words	50	0	Yes	General hospital care
Das et al (2023) [25]	Isolated words	8	0	N/A	Emergency situations
Ko et al (2019) [26]	Isolated words and sentences	419	105	Yes	Emergency situations
Barrientos-Villalta et al (2022) [27]	Isolated words	17	0	Yes	General hospital care
Ramírez Sánchez et al (2021) [28]	Isolated words and sentences	49	20	Yes	Primary care consultations
Gandhi et al (2021) [30]	Isolated words	20	0	Yes	General hospital care
Uchil et al (2020) [31]	Isolated words	20	0	Yes	General hospital care
Areeb et al (2022) [22]	Isolated words	8	0	N/A	Emergency situations
Adithya and Rajesh (2020) [36]	Isolated words	8	0	No	Emergency situations
Ihsan et al (2024) [37]	Isolated words	30	0	N/A	General hospital care
Das et al (2024) [38]	Isolated words	6	0	N/A	Primary care consultations
Faisal et al (2023) [39]	Isolated words	293	0	N/A	Primary care consultations
Bellil et al (2024) [42]	Isolated words	10	0	No	General hospital care
Sensor-based (depth-sensing)					
Hisham and Hamouda (2019) [29]	Isolated words	42	0	No	General hospital care
Sarhan et al (2015) [33]	Isolated words	16	0	No	Emergency consultations
Süzgün et al (2015) [35]	Isolated words	33	0	N/A	General hospital care
Dewasurendra et al (2020) [41]	Isolated words	0	N/A	No	Emergency situations
Sensor-based (glove-based)					
Deji Dere et al (2022) [32]	Isolated words	5	0	No	Emergency situations
Guo et al (2023) [34]	Isolated words	More than 550	0	N/A	Emergency situations
Luqman and Mahmoud (2020) [40]	Isolated words and sentences	3327 sign words and 30,296 singular words with their plurals	600	Yes	General hospital care
Hybrid					
Sosa-Jiménez et al (2022) [12]	Isolated words	43	0	Yes	Primary care consultations

a N/A: not applicable.

The system developed by Sosa-Jiménez et al [12] is the only one classified as hybrid, as it leverages Kinect’s red, green, blue, and depth sensors to recognize signs through both visual input and 3D skeletal tracking. Because it is the only hybrid system, it is presented separately in Table 1 and Multimedia Appendix 5; however, in the following sections, it will be discussed alongside the sensor-based systems (depth-sensing).

### Aspects Related to System Development

#### Languages Involved, Direction of Communication, and Capturing of Facial Expressions

##### Image-Based

The systems studied supported a range of spoken-sign language pairs, including Arabic (Algerian and Saudi), English, Chinese, Brazilian, Portuguese, Greek, Indian, Korean, Peruvian, and Mexican Spanish, and their respective sign languages (Table 2). Only 3 papers successfully implemented a bidirectional communication between spoken and sign languages [15,35,39], while another study attempted bidirectionality but did not achieve it [13].

Only 2 studies explicitly addressed structural and cultural differences between spoken and sign languages in their translation processes [15,28]. Additionally, 7 systems incorporated the recognition of facial expressions, facial key points, and other nonmanual features (such as audio phonemes and body posture) to enhance translation accuracy and communication effectiveness [15,26-28,30,31,39].

##### Sensor-Based

These systems also supported multiple spoken-sign language pairs, including combinations involving Arabic (and its Egyptian variant), English, Turkish, Sinhala, and Mexican Spanish, along with their respective national sign languages (Table 1).

In total, 3 studies reported successful implementation of bidirectional communication between spoken and sign languages [12,34,41]. One study took linguistic differences into account by applying rule-based morphological and syntactic processing to accommodate features specific to Arabic Sign Language—such as subject-initial word order, lack of inflection, and the use of spatial and visual cues—thus enabling transformation into grammatically correct Arabic sentences [40].

Two sensor-based systems also linked corpus construction to the use of facial expression and body movement capture for more precise recognition [12,32]. As anticipated, no glove-based system used such an approach.

### Corpus Used to Generate the Language Database for System Development

#### Image-Based

Overall, most systems relied on corpora composed of isolated words or terms, except for 3 studies, which included full sentences [14,26,28]. Two studies defined their corpus based on real-world contexts [15,26], while others relied on datasets designed by the authors [13,30], consulted health professionals [14], or extracted content from dictionaries [31]. Four systems focused on essential or emergency vocabulary, often including the alphabet, numbers, and isolated signs relevant to health care, obtained through internet searches or expert input [22,25,36].

#### Sensor-Based

One glove-based system developed its corpus through consultations with health professionals and internet searches [32], while another was based on real emergency call transcripts [34]. A third glove-based system included an extensive set of 600 health-related sentences covering various types (nominal, verbal, and questions) with over 3000 signs [40]. A fourth one focused on basic signs only (fingerspelling the alphabet, numbers, and isolated sign words) [32].

Among depth-sensing systems, 3 built their corpora using isolated words or terms [29,33], while others developed their corpora in consultation with health care professionals [12,35] or constructed its corpus using full sentences commonly used during emergency calls [41].

### Health Care Context

#### Image-Based

Among the 15 studies in this group, 46.7% (7/15) were based on the general hospital environment [13,15,27,30,31,37,42], 36.7% (4/15) focused on emergency services or situations [22,25,26,36], 20% (3/15) were based on primary care consultations [28,38,39], and 6.7% (1/15) on psychiatric interviews [14].

#### Sensor-Based

In total, 2 of the glove-based systems used emergency services or situations as a basis [32,34], and the remaining one used the general hospital environment [40], while 2 depth-sensing systems were based on care provided in a general hospital environment [29,35], 1 used the context of primary care consultations [12], and 2 used emergency services [33,41].

### Approaches and Techniques for System Development

#### Image-Based

In the image-based systems reviewed, different datasets have been created, involving capturing images in many lighting conditions and backgrounds, recording of signs made by users fluent in sign languages, extracting keyframes from videos [15,22,25-28,33-38], and image stacking [30]. These datasets have been divided into training, validation, and test sets for developing and evaluating the proposed systems [13,14].

Data processing included methods such as hierarchical classification, feature extraction through neural networks, human key point estimation, and pose, hand, and skeleton coordinates of people in videos [14,15,25,27]. Different models were used, such as CNNs, long short-term memory, hidden Markov model (HMM), OpenPose, 3D graph convolutional network, long-term recurrent convolutional network, 1D-CNN, and bidirectional long short-term memory, both individually and in combination, for the classification and prediction of sign language signals [15,22,26,27,30,37-39,42].

Additionally, only 1 paper presents an avatar module that transforms a hearing person’s text into sign language [39]. The system comprises 3 modules: a signal recognition module, a speech recognition and synthesis module, and an avatar module. Each module performs specific tasks to ensure the integrated functioning of the system.

#### Sensor-Based

In sensor-based systems, development methods involved capturing sign language signs with a Kinect, Leap Motion Controller, and cameras on wearable devices such as smart glasses [32-35,41]. Then, the captured data were processed and analyzed to extract relevant characteristics, such as position, movement, and shape of the hand; wrist trajectory; and other specific features of the sign [32,34].

Different techniques have been applied in the development of these systems, including machine learning models such as random forest, naïve Bayes classifier, Ada-Boosting, dynamic time warping, and HMMs [12,29,35]. Furthermore, preprocessing, segmentation, and feature extraction techniques have also been used to improve the effectiveness and accuracy of the systems in detecting and interpreting signs [32-34].

One of the systems also integrated machine translation processes by applying morphological and syntactic analysis to restructure sentence structure and ensure grammatical agreement [40].

### Technologies Involved

The technologies involved in system development are shown in Multimedia Appendix 6.

### The Infrastructure and Training Required for Use

#### Image-Based

Desktop and notebook computers, cameras, sensors, and supporting devices and equipment were needed to implement 3 proposed systems [25,27,36,42], whereas a mobile phone was required for 4 of them [27,30,31,42]. Beyond internet connectivity, 1 proposal [29] required downloading apps. In the image-based group, 2 systems explicitly mentioned using a phone’s camera [30,31], and 3 mentioned using a webcam or external camera [13,36,37]. None of the studies addressed the details of the training required for the translation system. In total, 3 papers failed to address the structure and training required for use [14,22,26].

#### Sensor-Based

Three systems, all depth sensing [29,33,35], addressed the need for a computer connected to a power source. Only 1 depth-sensing system pointed out the need for a mobile phone with an integrated camera [41]. One glove-based [34] proposal required downloading an app, and this was the only one that described the use of a phone’s camera, additionally requiring augmented reality glasses with a Bluetooth interface. Another one, from the depth-sensing type [33], mentioned the existence of an external camera device. Microsoft Kinect is a standard device for sensor-based systems that uses depth sensing [45]. None of the studies addressed the details of the training required for the translation system. One paper did not discuss the necessary structure and training for implementation [40].

### Multidisciplinary Partnerships

#### Image-Based

Various disciplines were represented among the research teams, who reported back to university departments of informatics [14,30], applied sciences [27,42], engineering [13,14,30,37,38], technology [26,30,31,37], computer science [22,38,39,42], and biomedical physics [37]. Only 4 studies mentioned the participation of linguistic specialists [13,15,26,32] during corpus development or database recording to improve sign language representation, and only 1 mentioned having involved physicians [14] in selecting the words to create the corpus. Along with sign language interpreters, hearing-impaired native sign language speakers [11,14,26] were engaged to record the videos.

#### Sensor-Based

The sensor-based systems encompassed a great variety of backgrounds, including university departments of statistics [12], AI [12,32], informatics [12,41], applied sciences [12], engineering [29,33,35], technology [33,41], and computer science [32,34,40]. To contribute to the enhancement of sign language representation, some systems involved linguistic specialists [35] in the development of a corpus or database, and also deaf native sign language speakers [32,34] and sign language interpreters worked on recording the videos. Physicians [12,32,35] and deaf individuals [35] were also involved in selecting the words for the corpus.

### Sample Size (Sample Size of Individuals to Develop the System, Users, and Content)

#### Image-Based

The sample size (number of individuals) used to develop the systems ranged from 2 [31] to 26 [22,25,36] among systems that used the same people for development and testing, and 1 study did not inform the sample size used in the development phase [38]. For systems that used different individuals for development and testing, the number used in the development phase ranged from 14 [26] to 33 [39], and the number of individuals used in the test phase ranged from 2 [39] to 6 [26]. Four studies [27,28,30,42] did not mention whether they used the same individuals for both development and testing. The dataset’s contents ranged from 21 scripts [14] to 145,035 videos (Table 2) [39].

#### Sensor-Based

The sample size (number of individuals) used to develop the systems ranged from 2 [32,40] to 6 [34] among systems that used the same people for development and testing. For systems that used different people for development and testing, the number of people to develop ranged from 2 [40] to 12 [12] (2 studies [33,46] did not inform the sample size in development), and the number of users to test ranged from 2 [39] to 10 [29].

The content of the datasets ranged from 10 videos (5 for the training phase and 5 for the testing phase) [32] to 1260 samples (840 samples for the training set and 420 samples for the testing set) [29]. In total, 2 studies did not inform the size of the content of their datasets (Table 2) [40,41].

### Cost

#### Image-Based

None of the included studies provided quantitative data on the costs associated with the development of their SLR systems, either in terms of technological infrastructure or human resources.

#### Sensor-Based

As with image-based studies, no quantitative analysis has been reported regarding the costs for developing the SLR systems. However, one of the studies mentioned that the development cost of the device “SmartCall” was much less when compared to those of similar works (which used sensors embedded in smartwatches), suggesting that researchers in low- and middle-income countries could readily prototype their own devices [32].

### Testing

#### Image-Based

The tests have been conducted in simulated environments involving different training networks, varying noise levels, and different scenarios [13,14]. Data splitting strategies into training and testing sets [15,22,25,36-39] and comparing different methods [18] have been used to evaluate the accuracy and effectiveness of the developed systems in detecting and interpreting signs.

Accuracy measures in detecting and interpreting signs ranged from 25% [27] to 100% [42]. Precision ranged from 91.5% [25] to 100% [42] (5 systems) [22,24,25,37,42]. Recall ranged from 90.1% [25] to 100% [42] (4 systems) [24,25,37,42]. *F*_1_-score ranged from 90.7% [25] to 100% [42] (4 systems) [24,25,37,42]. No study presented specificity and sensitivity (Multimedia Appendix 5).

A common difficulty was the failure of some systems to predict similar hand movements, given the proximity of the key points [22,30,31]. For that reason, double-handed signs were more accurately classified than single-handed ones [22].

#### Sensor-Based

The tests were conducted using datasets, in which different users performed sign language signs repeatedly in different scenarios [12,32,35]. Testing also involved splitting data into training, validation, and test sets and evaluating the performance of the systems [29,32,33]. Additionally, usability and user acceptance tests have been conducted to assess the effectiveness and practicality of the systems in real-life situations [34,35].

Accuracy measures in detecting and interpreting signs ranged from 80.5% [33] to 99.8% [41] (8 systems) [12,29,32-35,40,41], being 80.5% [33] to 99.8% [41] for systems with depth-sensing sensors and 72% [32] to 92% [40] for systems with hand glove sensors. Only 1 study [12] presented precision (“>90%”). Recall ranged from 90.1% [25] to 98.8% [24] (3 systems) [24,25,37]. *F*_1_-score ranged from 85% [32] to 88.6% [12] (2 systems) [12,32]. Only 1 study [12] presented specificity and sensitivity (99.8% and 87.9%, respectively; Multimedia Appendix 5).

### Evaluation or Use in Real Context

#### Image-Based

Among image-based studies, authors of 11 of the studies [13-15,22,25-28,31,36,38,39] did not respond to the emails sent by the research team, so it was impossible to update the current status of their systems. Authors of 2 of the studies informed that their systems have not been implemented in real scenarios or commercial settings [30,42]. One author mentioned that his team is in the process of developing a smartphone app for interpreting medical signs and that they will proceed to real-world implementation once it is ready [37].

#### Sensor-Based

Among sensor-based studies, the authors of 3 of the studies [29,33,41] did not respond to the emails sent by the research team, so it was not possible to update the current status of their systems. Authors of 4 other studies informed that their systems have never been deployed or commercialized [12,34,35,40]. Authors of the other study announced that their system is currently undergoing major design improvements and patent applications and that they hope to make it commercial very soon [32]. Authors of one of the studies reported that they open-sourced their dataset and their source code implementation to facilitate adoption of their system and accelerate technology transfer among research groups [32].

### Emergency Responses Capability

Regarding emergency circumstances, 13 studies [12-15,22,25,26,28,30,32,33,35,36] analyzed the capabilities of the system to respond in urgent situations according to various parameters.

#### Image-Based

Several image-based systems assessed key performance indicators relevant to urgent health care contexts, including recognition speed for signs and phrases [12,13], the system’s ability to reliably alert health care providers [12,14,34], and the clarity of communication output [12,26]. Some studies also addressed design considerations critical for emergency use, such as error tolerance (the system’s resilience to technical failures) [25], and the inclusion of medical vocabulary specific to emergency scenarios [30]. One system also incorporated structured protocols aimed at guiding interactions with deaf patients during emergencies [15]. However, a common limitation was the small size of the vocabulary recognized, which restricted the ability to handle diverse emergency dialogues [22,36].

#### Sensor-Based

Sensor-based systems also demonstrated potential for real-time emergency support. For instance, one system was specifically designed to improve access to emergency services through depth-sensing technology [41], while another emphasized robustness through error-tolerant functionality [12]. Similar to image-based tools, sensor-based systems faced limitations related to the restricted number of medical terms recognized, which could hinder effective communication during critical situations [32,34].

### Technological Characteristics

#### Image-Based

None of the studies reported on critical aspects such as system reliability (graceful degradation or recovery after crashes), data security, video storage, or deletion policies after translation, or objective metrics comparing communication outcomes with and without the system. Translation time was superficially addressed in 1 paper [22]. Authors compared 3 different models (a 3D CNN; a pretrained visual geometry group 16 layers and a recurrent neural network with a long short-term memory scheme; and a model based on YOLO v5, an advanced object detection algorithm). When comparing the proposed techniques, the YOLO-based model was faster, with an image processing rate of 40‐90 frames per second. Therefore, it could be used for emergency sign recognition with only a few milliseconds of delay.

#### Sensor-Based

Only 3 studies addressed translation time in sensor-based systems [29,34,41]. One study compared different machine learning classifiers and reported the following processing times: 8.09 seconds for the naïve Bayes classifier, 15.3 seconds for random forest, 17.63 seconds for dynamic time warping, and 20.8 seconds for HMM [29]. Another system reported an average latency of 0.55 seconds for translating 550 signs, with mobile translation taking 122 milliseconds and phoneme streaming 206 milliseconds to render the animation [34]. The third study reported that the system required 3 to 4 seconds from the end of the gesture to generate the output. In test scenarios, it consistently outperformed Google Cloud, with response times of 2.5, 2.6, and 2.2 seconds, compared to 4.1, 4.2, and 3.7 seconds for Google Cloud [41].

As with image-based systems, none of the sensor-based systems discussed reliability, data privacy, postprocessing video storage, or communication effectiveness metrics.

### User Experience

#### Image-Based

User experience was not explicitly evaluated in any of the image-based studies. A few of them commented on some factors related to the usability of the developed systems, both about their facilities and difficulties. These feedbacks are listed in Multimedia Appendix 5.

#### Sensor-Based

The evaluation of users’ experience was subjectively reported in 4 sensor-based studies [34,35,40,41]. The first one, classified as glove-based, conducted a user study on the signers’ experience. The study involved 12 participants, and Quality of Experience was rated on a 5-point scale, ranging from 0=worst evaluation)to 5=best evaluation, to assess the usefulness of the system. The obtained results were 4.2 for accessibility, 4.3 for usability, and 4.6 for overall experience [34].

Additionally, a second study, classified as depth-sensing, developed a questionnaire score using the same scale for 5 statements. In total, 5 participants used the system, and afterward, responded to the questionnaire. The questionnaire results obtained for each statement question were 4.85 for “I was able to express my situation using HospiSign,” 4.85 for “HospiSign is easy to use,” 5.00 for “Most people could learn to use HospiSign quickly,” 4.45 for “Options were sufficiently clear,” and 5.00 for “HospiSign would help the hearing impaired” [35].

Another study, from the glove-based category, provided a manual evaluation performed by 3 native Arabic speakers. Each of the 600 sentences was classified by them as understandable, somehow understandable, and not understandable according to grammatical and semantic metrics. The respective results were 80%, 12% and 8%, demonstrating an acceptable translation provision in 92% of the sample [40].

Additionally, a third study, classified as depth-sensing, conducted a user satisfaction survey with 10 users, and the results were classified as high, medium, and low satisfaction. The results reported were user registration: high, text-to-call: high, call-to-sign: medium, sign-to-call: medium, and prerecorded message: high [41].

Finally, a fourth study, classified as depth-sensing as well, developed a questionnaire score using the same scale for 5 statements. In total, 5 participants used the system, and afterward, responded to the questionnaire, and the results obtained for each statement were 4.85 for “I was able to express my situation using HospiSign,” 4.85 for “HospiSign is easy to use,” 5.00 for “Most people could learn to use HospiSign quickly,” 4.45 for “Options were sufficiently clear,” and 5.00 for “HospiSign would help the hearing impaired” [35].

There were also some comments on the usability of the developed systems, concerning the user experience. These feedbacks are listed in Multimedia Appendix 5.

### Ethical Issues Addressed

Ethical concerns related to the use of communication systems for deaf and hard-of-hearing individuals were largely underexplored across the included studies. Importantly, none of the included studies discussed broader ethical implications of the systems themselves, such as data privacy and security, the handling and potential storage of video or biometric information, or the autonomy and psychological impact of using these technologies. Despite their role in sensitive health care contexts, no system reported design strategies for ensuring user dignity, minimizing potential emotional distress, or addressing users’ preferences regarding data management. Additionally, none of the studies evaluated whether addressing ethical aspects influenced system usability or user trust, including transparency, control over personal data, and informed decision-making.

## Discussion

### Principal Findings

The development of SLR systems represents a promising step toward improving communication between deaf individuals and health care providers. Despite the vast literature in SLR systems, many computer science studies focus on creating algorithms for recognizing (and less often translating) signed content [1]. These teams often lack deaf individuals with firsthand experience of the challenges the technology aims to address, and they may not fully understand the linguistic complexities of the language. Additionally, the algorithms are typically trained on datasets that do not reflect real-world scenarios, making these 1D approaches to sign language processing of limited practical value [1]. In our analysis, the comprehensive search resulted in over 21,000 papers, but only 23 studies met our inclusion criteria. All analyzed systems are in the development and testing stage, with no real application yet, and this review highlights significant challenges that hinder the implementation of these systems in real-world settings. These challenges include variability in methodologies, limitations in dataset quality, and the underrepresentation of key communication elements, such as bidirectionality and facial expressions. Furthermore, the absence of cost analysis and the lack of multilingual integration reflect gaps in the current body of research. To address these issues, it is essential to evaluate both the technical aspects and the practical implications of SLR systems, aiming for inclusive and effective solutions.

Image-based systems accounted for the majority of the studies. This predominance highlights the versatility of image-based systems, which capture facial expressions, head movements, lip reading, and hand gestures using simple devices like mobile phone cameras [47]. In contrast, it is possible to infer that sensor-based systems, which rely on advanced technologies such as depth-sensing cameras and sensor-embedded gloves, incur higher costs [48]. However, a critical gap identified across studies was the lack of clear cost-related information. Only 1 study briefly mentioned that its device, “SmartCall,” was more affordable compared to similar solutions, suggesting its feasibility for low- and middle-income countries [32].

Given the lack of sufficient cost-related data in the studies reviewed, it would be challenging to provide accurate inferences regarding the cost-effectiveness of these technologies. Furthermore, as costs vary significantly depending on the country and context, it would be difficult to generalize these findings across all regions. This is a notable limitation, and we believe that future studies should address this gap by providing more detailed and context-specific cost analyses.

### Corpus Definition and Number of Samples

Corpus definition and dataset size were highly heterogeneous across the reviewed studies, reflecting diverse development contexts and objectives. Most systems relied on isolated words, limiting applicability in real-world health care communication, while some incorporated phrases or real clinical contexts, occasionally guided by health professionals or dictionaries [14,26,28,40]. A few image-based systems also integrated facial expressions and body movements, enhancing linguistic representation [15,26-28,30,31,39]. Among sensor-based systems, corpus construction lacked multimodal features, particularly in glove-based systems, which are limited in capturing facial and body expressions.

Sample sizes ranged widely from as few as 10 to over 145,000 videos, raising concerns about the adequacy of data for training reliable models. This lack of standardization in corpus design and dataset size limits the comparability, scalability, and generalizability of SLR systems in clinical settings. Establishing minimum standards for corpus development could improve system robustness and support broader implementation in health care.

### Key Aspects of Effective Communication

Bidirectionality and facial expression recognition are 2 critical components for effective communication, but were not included in the majority of publications. Most systems were unidirectional, translating only from sign language to written text, impairing its applicability in real-world contexts. While such systems may enable health care practitioners to understand what a deaf patient is communicating, they fail to facilitate communication in the opposite direction, thereby undermining true interactive dialogue.

Facial expression recognition is a vital element for capturing linguistic prosody, and although face landmarks were considered in 8 papers [15,26-28,30,31,39,40], only 1 of them explicitly addressed it as an indicator of grammatical tense [28]. This omission hinders the natural and nuanced communication required in health care contexts.

Notably, only 3 studies explicitly acknowledged the cultural and structural distinctions between sign languages and oral languages [15,28,40]. This omission is significant, as sign languages are not mere gestural representations of spoken language, but complete linguistic systems with their own grammar, syntax, and cultural context. Failure to consider these differences can lead to inaccurate or overly literal translations, potentially compromising communication quality and user trust in assistive systems. The limited attention to these linguistic specificities suggests that many of the reviewed systems may have been developed from a technical standpoint, with insufficient involvement of deaf communities or sign language experts.

Given the numerous challenges involved, it is nearly impossible to develop SLR tools that achieve 100% accuracy with a large vocabulary [49]. For example, the same sign can have large changes in shape when it is in different locations in the sentence [50]. In the studies analyzed, only 1 demonstrated perfect accuracy (100%), but this was based on an image-based system, which was tested only with isolated words [12]. Studies that involved a relevant number of sentences and words tended to perform worse, with accuracy sometimes too low to enable effective communication [14,26].

### Multilingual Challenges

Sign languages are not universal, and they are not mutually intelligible, and they can present significant differences between countries, and sometimes even within the same country [51]. While some studies proposed extending their systems to multiple languages [12,28,41], none successfully implemented multilingual translation. The inherent diversity of sign languages—each with unique grammar, vocabulary, and body configurations—poses substantial challenges to creating universal systems [27,52,53]. Furthermore, there was a predominance of English corpora [25,30-32,34,36,37]. This is probably driven by high research investments, as well as the global reach and accessibility of English [54], which not only facilitates better comprehension within the medical academic community but also enables broader dissemination and open sharing of the corpus [55]. This indicates a gap in accessibility for non-English–speaking regions, emphasizing the importance of expanding datasets to include diverse languages and cultures. To address this gap, it is crucial to foster collaboration between researchers from different countries, sharing sign language corpora and visual data, so that a truly multilingual system can be developed.

### Multidisciplinary Partnerships

As Bragg et al [1] described, an interdisciplinary approach is crucial to sign language processing. Representatives of the deaf community and health care practitioners must be involved in the team to better understand the needs of the community and the specific purposes for which the technology is intended. Linguistics plays a key role in identifying the structures of sign languages that the algorithms need to process. Natural language processing and machine translation offer valuable techniques for modeling, analyzing, and translating. Computer vision is necessary for recognizing signed content, while computer graphics are essential for generating it. Finally, human-computer interaction and design are vital for developing comprehensive systems that meet the needs of the community and integrate seamlessly into people’s daily lives [1].

The inclusion of multidisciplinary teams was a notable feature in several studies, combining expertise from linguistics, inclusive design, AI, and health care practitioners. However, the participation of deaf community representatives was limited, potentially undermining cultural and linguistic relevance.

### Ethical and Data Security Concerns

As these systems are intended to support real-time communication in clinical settings, their design must go beyond technical optimization to consider ethical issues such as privacy, autonomy, and emotional safety, dimensions largely overlooked in the reviewed studies. The reviewed literature does not adequately address the psychological impact of using these systems. Issues such as the psychological effects on users, especially those who are deaf or hard of hearing, were not discussed in any of the studies. These concerns are particularly important, as assistive technology should promote autonomy and well-being without causing additional stress or emotional burdens for users [56].

Data security is another critical concern that received little attention. Many SLR systems collect sensitive information, such as facial images, gestures, and body movements, which may contain identifiable or private data. However, few studies addressed how these data are stored, protected, or deleted. In health care contexts—where confidentiality is paramount—this lack of attention to privacy and data protection represents a significant oversight.

These omissions highlight a critical gap: as assistive communication technologies are implemented in real-world health care settings, particularly among structurally marginalized populations like the deaf community, ethical design and responsible implementation are essential. Future studies should incorporate ethical considerations from the early stages of system development and report these aspects transparently.

### User Experience

Overall, few studies provided reports on the users’ experience with the developed systems, which were structurally evaluated and described in only 2 of them [22,36], and only with a small number of participants (12 in Areeb et al [22] and 5 in Adithya and Rajesh [36]). The other studies focused on usability metrics of the developed systems [12,14,15,25,29]. The issues raised corroborate difficulties described in the literature. All recognition systems face the challenge of hand tracking and struggle with self-occlusion between fingers, with additional difficulties in single-hand signals [57]. Such an aspect was mentioned especially by the authors of image-based systems, since in their approaches they did not attempt 3D perception to mitigate occlusion [14,15]. Furthermore, another significant obstacle is the interpersonal variation between signers, which includes not only their physical characteristics but also the way they perform gestures. Minor differences in signaling are important constraints for AI and recognition tools, justifying the reports regardless of the system’s type [12,26].

Although the studies that presented user evaluation [34,35] reported that the systems performed well, showing positive impacts on accessibility and effective communication, the evidence is limited, as only 2 studies were conducted, and both involved a small number of users. User experience goes beyond the system’s accuracy and usability, and according to International Organization for Standardization [58], it refers to a “person’s perceptions and responses resulting from the use and/or anticipated use of a product, system or service,” including “all the users’ emotions, beliefs, preferences, perceptions, physical and psychological responses, behaviours and accomplishments that occur before, during and after use.” Thus, generating a positive user experience for the deaf users is essential for the adoption and use of the proposed systems. While studies typically focus on accuracy and usability metrics, it is equally important to evaluate other factors, such as learning cost and response speed, which can significantly affect user adoption, especially in health care contexts, where ease of use and time efficiency are critical.

Saeed et al [59], in a review on system-based sensory gloves for SLR, reported that user comfort was a challenge, as using a sensory glove required the user to wear a bulky glove containing sensors, cables, and a circuit board, which limited the user’s hand mobility. However, we believe this issue is gradually being reduced, as sensors continue to evolve, becoming smaller and more efficient. Furthermore, the ability to connect these sensors via Bluetooth has further minimized the bulkiness, offering more comfort and flexibility for the user.

The design of interfaces for automatic sign language translation systems, specifically their integration within web-based and social media applications, necessitates careful consideration of numerous factors. Research conducted by Debevc et al [46] and Kožuh et al [60] addresses methodologies for enhancing the interaction experience for deaf and hard-of-hearing users within digital communication platforms [46,60]. The authors examine methods to improve accessibility [46,60].

A recurring concern highlighted in these studies is the potential for picture-in-picture windows to disrupt the user experience for nondeaf individuals. Therefore, the implementation of picture-in-picture displays should be judiciously considered and minimized. The domain of automatic sign language translation is characterized by rapid advancements and multifaceted complexities. The techniques used to optimize the user experience for these systems, while of significant relevance, warrant a dedicated review to comprehensively assess their efficacy and implications.

### Limitations and Strengths of the Review

This review is primarily qualitative, as the diversity in methodologies, technologies, and parameter calculations precluded quantitative synthesis. Additionally, there are no standardized tools to assess the risk of bias in these types of studies included. Furthermore, this review focused on systems that recognize sign language. Although these systems do not fully represent the portion of the deaf community that prefers lip reading, writing, or other methods of communication, they do include individuals who use sign language. Finally, 4 publications could not be included, as they were not retrieved despite at least 3 contact attempts regarding 2 studies and due to the unavailability of authors’ contact details of the other 2 studies.

The strengths of this systematic review include prospective registration and publication of a protocol [18,19]. Furthermore, it adheres to the Cochrane Guidelines and the PRISMA statement [20,21], and it has a broad search strategy encompassing multiple languages and publication types. A multidisciplinary team, including a deaf linguist, ensured diverse perspectives and representation.

Additionally, this study is innovative, as it focuses specifically on SLR for the health context, an area in which social inclusion and understanding differences are extremely important. Although other reviews were previously published, the majority of them cannot be considered true systematic reviews [49,59,61,62]. They lack a prepublication of a protocol, a robust methodology, a broad search strategy, paired screening and data extraction, and comprehensiveness. They usually focus on technical aspects of a broader and more general analysis of sign language translation systems and use restricted publication periods and language (English only) in the search. Overall, they did not include a search in MEDLINE, which is a crucial database for health-related research, and presented methodological shortcomings, such as not mentioning the use of paired reviewers for study selection and data extraction. Furthermore, they have not reported extracting data on user experience, cost, and data security. Unlike these more general reviews, our study is unique in focusing on the health care context. Therefore, our study fills these gaps, providing a more robust, detailed, and comprehensive contribution specifically to the health field.

### Final Considerations

Image-based systems dominated the field, demonstrating greater accessibility but facing challenges with lighting conditions and environmental variability. While sensor-based systems showed higher precision, their reliance on specialized equipment limits scalability. Critical aspects, such as bidirectional communication and facial expression recognition, remain underexplored, hindering the practical implementation of these systems in health care settings.

Based on the evidence reviewed, effective SRL systems for real-world health care scenarios are scarce. Future research should focus on developing adaptive systems capable of recognizing diverse sign languages and addressing the varying communication needs of the deaf community. It should prioritize inclusive and participatory design processes that respect the linguistic integrity of sign languages and address sociocultural nuances critical to effective communication. Expanding datasets, incorporating user feedback, and fostering multidisciplinary collaborations will be pivotal for creating inclusive and scalable solutions.

Additionally, future research should incorporate a comprehensive assessment of the proposed systems, addressing both technical and user-centered aspects. This assessment should consider device compatibility (especially for smartphone-based systems), required learning time, translation accuracy across different signers and accents [63], user trust in automated translation, its effect on self-expression, and privacy implications. These evaluations should not only be conducted once the system is fully implemented but also throughout the development process (ie, formative evaluation) [64].

Another significant gap identified in the literature is the lack of a standardized evaluation framework for SLR systems. The inclusion of consistent and well-defined indicators, such as user satisfaction and economic viability, would provide a more comprehensive understanding of the systems’ effectiveness and their potential for widespread adoption. User satisfaction is a crucial success factor, and including economic indicators would help assess whether the systems can be feasibly adopted in different settings, especially in low- and middle-income countries. The implementation of the framework would not only enhance the comparability of findings but also provide valuable insights for policymakers and stakeholders in the health care industry.

Furthermore, we suggest that the development of future SLR solutions involves experts in ethics, law, and data security to ensure compliance with legal and ethical standards and to protect users during the implementation of these technologies. Studies should provide more detailed information about how ethical and data security issues are managed and the potential impacts on user trust and adoption.

One notable strength identified in this review was the initiative of a research group to open-source both their dataset and system code [32]. This practice promotes transparency, reproducibility, and collaboration—key elements for the advancement of SLR systems. By allowing other researchers to access and build upon existing work, open data and open-source tools can accelerate the refinement of models and the development of more accurate and user-friendly solutions. In the long term, such collaborative practices may contribute to the creation of robust, scalable systems that are truly usable in health care settings, bridging communication gaps and improving care for deaf individuals. Encouraging the adoption of open science principles within the SLR research community could significantly enhance innovation and standardization efforts in this emerging field.

### Conclusions

This review highlights the ongoing development of communication systems designed to assist deaf individuals who use sign language to improve their interaction with health care providers. There was a predominance of image-based approaches over sensor-based ones, though both have demonstrated substantial variability in accuracy in recognizing and interpreting signs. However, critical aspects such as bidirectional communication and the recognition of facial expressions, which are essential for effective communication, were notably absent in most studies. None of the systems has reported to have addressed all aspects critical to integration into health care settings. These findings underscore the need for further research, especially regarding the practical implementation of these systems, their usability, and their overall effect on the quality of care for deaf patients.

## Supplementary material

10.2196/70417 Multimedia Appendix 1 Search strategy.

10.2196/70417 Multimedia Appendix 2 The definitions used regarding the type of sign language recognition system, the corpus formation, and the health context.

10.2196/70417 Multimedia Appendix 3 Codebook.

10.2196/70417 Multimedia Appendix 4 Studies not retrieved.

10.2196/70417 Multimedia Appendix 5 Development and testing of different sign language recognition systems.

10.2196/70417 Multimedia Appendix 6 Technologies necessary for sign language recognition system implementation. 1, 2, 3

10.2196/70417 Checklist 1 PRISMA checklist.

## Abbreviations

AI: artificial intelligence
CNN: convolutional neural network
HMM: hidden Markov model
PRISMA: Preferred Reporting Items for Systematic Reviews and Meta-Analyses
SLR: sign language recognition
YOLO: You Only Look Once
