# Supplementary material for: Innovations in Deaf Health Care Communication: Systematic Review of Sign Language Recognition Systems
Source: J Med Internet Res. 2026 Apr 9;28:e70417. doi: 10.2196/70417 (PMC13065231; doi:10.2196/70417)
Supplement: Multimedia Appendix 1 [file jmir-v28-e70417-s001.docx]

**Search strategy**

| Medline  ("Neural Networks, Computer"[Mesh] OR "Artificial Intelligence"[Mesh] OR "Biomedical Technology"[Mesh] OR "Communication Aids for Disabled"[Mesh] OR "Image Processing, Computer Assisted"[Mesh] OR "Machine Learning"[Mesh] OR "assistive technolog*") AND ("Sign Language"[Mesh] OR "Hearing Loss"[Mesh] OR "Persons With Hearing Impairments"[Mesh] OR "Communication Barriers"[Mesh] OR "Gestures"[Mesh]) |
| --- |
| IEEE, ACM  (“Assistive technology” OR “Artificial Intelligence” OR “Computational Intelligence” OR “Machine Intelligence” OR “Computer Reasoning” OR “Computer Vision System” OR “Knowledge Acquisition” OR “Machine Intelligence” OR “Machine Learning” OR “Deep Learning” OR “Computer Neural Network” OR “Neural Network Model” OR “Perceptron” OR “Connectionist Model” OR “Neural Network” OR “Communication Aid for Disabled” OR “Speech Synthesizer” OR “Biomedical Technology” OR “Health Technology” OR “Health Care Technology” OR “Image Processing” OR “Computer-Assisted Image Analysis” OR “Computer Assisted Image Analysis”) AND (“Sign Language” OR “Hearing Loss” OR “Deafness” OR “Deaf”) |
| Web of Science, Google Scholar  (“Assistive technolog*” OR “Artificial Intelligence” OR “Computational Intelligence” OR “Machine Intelligence” OR “Computer Reasoning” OR “Computer Vision System*” OR “Knowledge Acquisition” OR “Machine Intelligence” OR “Machine Learning” OR “Deep Learning” OR “Computer Neural Network*” OR “Neural Network Model*” OR “Perceptron*” OR “Connectionist Model*” OR “Neural Network*” OR “Communication Aid* for Disabled” OR “Speech Synthesizer” OR “Biomedical Technolog*” OR “Health Technolog*” OR “Health Care Technolog*” OR “Image Processing” OR “Computer-Assisted Image Analys*” OR “Computer Assisted Image Analys*”) AND (“Sign Language” OR “Hearing Loss” OR “Deafness” OR “Deaf”) |
| Scopus  (“Artificial Intelligence” OR “Computational Intelligence” OR “Computer Vision System*” OR “Machine Learning” OR “Biomedical Technolog*” OR “Health Technolog*” OR “Health Care Technolog*” OR “Image Processing” OR “Computer- Assisted Image Analys*” OR “Computer Assisted Image Analys*” OR “sensor” OR “glove” OR “tactile” OR “haptics” OR “inertial” OR “bend sensor” OR “fiber optics") AND (“Sign Language translation”) |
