# Supplementary material for: Innovations in Deaf Health Care Communication: Systematic Review of Sign Language Recognition Systems
Source: J Med Internet Res. 2026 Apr 9;28:e70417. doi: 10.2196/70417 (PMC13065231; doi:10.2196/70417)
Supplement: Multimedia Appendix 2 [file jmir-v28-e70417-s002.docx]

1. Types of SLR system:

- 1. Image-based: systems that, utilizing computer vision techniques, analyze video input from cameras that capture hand gestures, facial expressions, and body movement [11,13,14].
  2. Sensor-based: systems that employ wearable devices equipped with sensors, such as accelerometers and gyroscopes, to capture hand and body movements, enabling the translation between signals into the spoken language [11,12,15].
     1. Glove-based: systems that utilize specialized gloves embedded with sensors, allow the capture of intricate motions, translating them into spoken or written language in real-time.
     2. Depth-sensor: systems that utilize depth-sensing devices, like Kinect or LiDAR technology, to capture 3D information about hand and body movements, enabling a precise recognition and interpretation of sign language gestures, by analyzing the depth and positioning of various body parts.
  3. Hybrid: image-based cameras are paired with other sensor types, like infrared depth sensors, which capture 3D information about hand and body movements, to gather multi-modal data, to improve the value of the vision data collected by using sensor readings [22,23].

2. Corpus construction:

- 1. Isolated words: words analyzed outside of any context or sentence, that is, only the central concept of the word is analyzed without considering nuances or variations that might arise in different contexts.
  2. Sentences: a unit of language composed of one or more groups of words that express a complete idea or statement. A sentence can be made up of isolated words that together convey a clear and complete message, with or without punctuation and other elements.

3. Healthcare contexts:

- 1. Primary care consultation: Consultations often available for the patient's first point of contact, offering comprehensive, accessible, community-based care. This level of care offers a spectrum of services ranging from health promotion and prevention to the treatment of acute and chronic diseases, palliative care, and rehabilitation.
  2. General hospital care: General hospital care is a set of services provided in a hospital environment, to promote adequate patient-centered care, diagnosis, and treatment for the patient, except in emergency situations.
  3. Emergency consultation: It corresponds to situations that require immediate medical intervention to guarantee the individual's well-being. These situations usually occur in ambulance and hospital environments.
  4. Emergency situation: It corresponds to attending to requests for help to guarantee the individual's well-being. These situations occur in out-of-hospital environments.
  5. Psychiatric interview: It consists of the interaction between health professionals and people with mental illnesses, to collect information about the individual's mental suffering.
