# Supplementary material for: Innovations in Deaf Health Care Communication: Systematic Review of Sign Language Recognition Systems
Source: J Med Internet Res. 2026 Apr 9;28:e70417. doi: 10.2196/70417 (PMC13065231; doi:10.2196/70417)
Supplement: Multimedia Appendix 3 [file jmir-v28-e70417-s003.docx]

**Codebook.**

| **Variable** | **Question** | **Label** |
| --- | --- | --- |
| Title | Title | Article title |
| DOI | DOI | Digital document identification |
| Author | First Author | 1st author of the article |
| Journal and year | Journal and year of publication | Journal in which it is published |
| Country | Country | The country where the study was implemented |
| Aim | Study Aim | Objective of the study presented in the article |
| Multidisciplinary partnerships | Multidisciplinary partnerships? Is there a specific framework for collaboration among the diverse fields? | Information about multidisciplinary partnerships (healthcare, technology, linguists, deaf people) and structures for collaboration between these areas |
|  | Multidisciplinary partnerships - corpus | Describe professional categories involved in the corpus |
|  | Multidisciplinary partnerships - system development | Describe professional categories involved in the development of the system |
| Deaf people as members of the team | Did the team have deaf people as members? | If the team has a deaf person(s) |
| Corpus examples | Corpus examples | Examples of terms used to develop the system |
| Language differences | Does it take oral/sign language differences into account when translating? | Information about the system considering the differences (syntactic, morphological) between oral and sign language |
| Image-based, Sensor-based or hybrid | Image-based, sensor-based or hybrid | Whether the system is image-based (uses only a standard camera to acquire images and analyze them), sensor-based (uses more developed sensors, such as gloves or more developed cameras) or hybrid |
| Communication system | Communication system development | Information about the development of the system |
| Communication system | Communication system intervention and testing | Information about the intervention (application) and testing (with people or videos of people) of the system |
| Languages involved | Languages involved (oral and sign) | Languages ​​involved in the translation (oral and sign languages) |
| Evaluation/Use | Evaluation/Use in real context (se tiver sido implementado) | Information about the use (and its evaluation) in a natural context |
| Users | Sample size (number of users) | Number of people who made signs to develop the system |
| Test users | Sample size (number of testes users) | Number of people who made signs to test the system |
| Content | Sample size (number of content) | Number of videos or signs used to develop the system |
| Health context | Health context (general, emergency, consultation) | Health context where the study was conducted (general, emergency, consultation) |
| Technology | Technology (hardware/software) necessary (for implementation) | Technology (hardware, software, and resources) required to implement the system in practice |
| Infrastructure and technology | Infrastructure and technology required for use (computers, electricity, internet) | Infrastructure and technology required for use (computers, electricity, internet) |
| Development technology | Development technology (artificial intelligence, image processing) | Technology used to develop the system (what was used to process the images) |
| Technology readiness level | Technology readiness level (development and testing or if it is being marketed) | Status of the technology (under development and testing or if it is being commercialized) |
| Translation time | Translation Time | Information on the time it takes to translate the signal |
| Confiability | Confiability (recovery after crashes) | Information on the reliability of the system, in the sense of its ability to reestablish itself after malfunctions |
| Data safety | Data safety (deletion/employment of videos after translation) | Information on the security of the data used, such as the deletion of users' videos after their signals have been translated |
| User experience (Patient experience) | User experience (patient): factors related to usability, such as ease/difficulty of using the system | Information on the user experience (patient) and factors related to usability, such as ease/difficulty of using the system, generally verified by questionnaire or evaluations |
| User experience (health professional experience) | User experience (health professional): factors related to usability, such as ease/difficulty of using the system | Information on the user experience (healthcare professional) and factors related to usability, such as ease/difficulty of using the system, generally verified by questionnaire or evaluation |
| Communication assessment | Measures (assessment) of communication improvement (with the system versus without the system) | Information about comparisons of communication with and without the system |
| Cost | Cost | Information about the cost of the system |
| Communication metrics | Communication-Related Evaluation Metrics | Assessment measures used for aspects related to communication |
| Evaluation Metrics | Subjective or Objective Evaluation Metrics | Subjective (directly related to the user) or objective (not directly related to the user, such as accuracy) assessment measures used |
| Accuracy measures | Accuracy measures | Accuracy measures (accuracy, sensitivity, specificity, precision, F1 score, etc.) for testing the system |
| Effectiveness communication | Communication Effectiveness (deaf person and doctor were able to understand communication?) | Information on the effectiveness of communication (were the deaf person and the doctor able to understand the communication?), generally assessed by questionnaire or assessment |
| Communication problems | Problems Reported in Communication | Problems reported in communication |
| Communication effectiveness | Criteria related to the effectiveness of communication (related to the demands of deaf people) - Is it bidirectional? Does it capture facial expression? | Information on criteria related to communication effectiveness (related to the demands of deaf people) - Is it bidirectional? Does it capture facial expressions? |
| Training | Training required to use the system | Information on the need for training of the team that will use the system |
| Patient privacy | Patient privacy | Information on patient privacy when using the system |
| System reliability and robustness | System reliability and robustness (reported crashes and recovery after crashes) | Information about the reliability and robustness of the system, i.e., whether the system has experienced failures and whether it has recovered after these failures |
| Age requirements | Age-specific requirements | Information about the age limits of users with which the system works or works best |
| Emergency response | Emergency response capabilities | Information about the system's ability to act in emergencies |
| Support for several languages | Support for several languages? | Information about whether the system can translate (to and from) different languages ​​(spoken and signed) |
| Input for system enhancement | Input for system enhancement? | Information about inputting data to improve the system |
| Deaf person | Is there a deaf person in the team? | Whether the team includes deaf person(s) |
| Ethical issues | Ethical issues addressed | Information about ethical issues addressed |
| Independence in healthcare management | Increased independence in healthcare management (reducing the need for intermediaries such as translators) | Information about increasing user independence in the health context related to reducing the need for intermediaries in communication, such as interpreters |
| Psychological Effects | Psychological Effects | Information about the psychological effects of the system on users |
| Real context | If the system has been implemented in real context, with real patients anywhere (Informação vem dos emails enviados para os autores dos artigos incluídos) | Information about the implementation of the system in a natural context, with actual patients (Information comes from emails sent to the authors of the included articles) |
| After implementation | Data after implementation about usability, user experience etc. (Information comes from emails sent to the authors of the included articles) | Information about the data obtained after the implementation of the system in a natural context, with actual patients (Information comes from emails sent to the authors of the included articles) |
| System availability | If the system is commercially available (Information comes from emails sent to the authors of the included articles) | Information about the commercial availability of the system (Information comes from emails sent to the authors of the included articles) |
