# Supplementary material for: Innovations in Deaf Health Care Communication: Systematic Review of Sign Language Recognition Systems
Source: J Med Internet Res. 2026 Apr 9;28:e70417. doi: 10.2196/70417 (PMC13065231; doi:10.2196/70417)
Supplement: Multimedia Appendix 4 [file jmir-v28-e70417-s004.docx]

**Studies not retrieved.**

| Article | Reason |
| --- | --- |
| Mangold H. Principles of automatic processing of speech signals and their application in medical technology and for aids for handicapped. Med Prog Technol. 1988;14(1):39-56. PMID: 2976877 | Unanswered contact attempts |
| McGraw S, Williams T. Physicians must provide auxiliary aids/services to assure effective communication with hearing impaired patients. Mich Med. 1995 Aug;94(8):10-1. PMID: 7565160 | Unanswered contact attempts |
| Plunkett LR. When the patient is hearing-impaired. N Y State Dent J. 2002 Jan;68(1):6-7. PMID: 11890010 | Authors’ contact not found |
| Astriani MS, Alvianto M. Telemedicine sign language classification for COVID-19 patients with disability based on LSTM model. J Eng Sci Technol. 2023;18(Special Issue):44-57. | Authors’ contact not found |
| Zizoune, Aicha & Zizoune, Asmae & Hamdaoui, Roba & Ziti, Soumia & Salah-Eddine, Karima & Kharmoum, Nassim & Anouar, Riadsolh. (2024). Real-Time Implementation of an AI-Based Virtual Sign Language Recognition and Interpretation System. 453-464. | Unanswered contact attempts |
| Karim, Tajbia & Mahayuddin, Zainal. (2023). Real-Time Bangla Sign Words Interpretation Uttered at Medical Emergencies. | Unanswered contact attempts |
