# Supplementary material for: Innovations in Deaf Health Care Communication: Systematic Review of Sign Language Recognition Systems
Source: J Med Internet Res. 2026 Apr 9;28:e70417. doi: 10.2196/70417 (PMC13065231; doi:10.2196/70417)
Supplement: Multimedia Appendix 5 [file jmir-v28-e70417-s005.docx]

Development and testing of different sign language recognition systems.

|  | **Sample size - development** | | **Sample size - testing** | | **Performance metrics** | | | | | |
| --- | --- | --- | --- | --- | --- | --- | --- | --- | --- | --- |
|  | **People** | **Number of content** | **Users** | **Sample for testing and development from the same dataset?** | **Accuracy** | **Precision** | **Recall** | **F1** | **Specificity** | **Sensitivity** |
| **Image-based** | | | | | | | | | | |
| Xia et al, 2022 [13] | 10 | More than 4,000 annotated SL images, were divided into the training set, validation set, and test set (3:1:1) | 10 | Yes (20% of the dataset) | 97% | NA | NA | NA | NA | NA |
| Pikoulis et al, 2022 [14] | 8 | 21 scripts, each one of them signed by each user | 8 | Yes | 60 - 70% | NA | NA | NA | NA | NA |
| da Silva et al, 2024, [15] | 10 | 5,000 videos (each user performed each sign 10 times): 70% used for training, 20% forvalidation and 10% for testing | 10 | Yes (10% of the dataset) | I3D (RGB) [15]: 95.32%  I3D (Flow) [15]: 95.31%  2-Stream I3D [15]: 96.12%  Image + Flow + Body Skeleton [41]: 98.80% | I3D (RGB) [15]: 94%  I3D (Flow) [15]: 96%  2-Stream I3D [15]: 95%  Image + Flow + Body Skeleton [41]: 99% | I3D (RGB) [15]: 95.32%  I3D (Flow) [15]: 95%  2-Stream I3D^8^: 96%  Image + Flow + Body Skeleton [41]: 98.80% | I3D (RGB)^8^:94%  I3D (Flow) [15]: 95%  2-Stream I3D [15]: 95%  Image + Flow + Body Skeleton [41]: 98.78% | NA | NA |
| Das et al, 2023 [25] | 26 | 412 videos: 50% used for training and 50% for testing | 26 | Yes (50% of dataset) | CNN (VGG-19): 90.29%  HM: 93.68%  ZM: 94.17% | CNN (VGG-19): 91.5%  HM: 94%  ZM: 94.5% | CNN (VGG-19): 90.12%  HM: 93.62%  ZM: 94% | CNN (VGG-19): 90.75%  HM: 93.37%  ZM: 94.12% | NA | NA |
| Ko et al, 2019 [26] | 14 (10 for the training phase and 4 for the testing phase) | 14,672 videos: 10,480 were used for training/development and 4,192 for testing | 6 | No | Sentence-Level: 45-56%  Gloss-Level: 55.28% | NA | NA | NA | NA | NA |
| Barrientos-Villalta et al, 2022 [27] | 8 | 439 videos^c^ | NA | NA | No filter: 25%  Wiener filter: 26%  Median filter:  28%  Wiener + Median filter:  27% | NA | NA | NA | NA | NA |
| Ramirez Sanchez et al, 2021 [28] | 6 | An average of 35 samples was collected for each one of the 49 signs (13 verbs and 36 words) and 15 samples for each facial expression across the three verb tenses (past, present, and future)^b^ | NA | NA | Focus on isolated words: 94.9% ± 0.07  Focus on sentences: 94.1% ± 0.09 | NA | NA | NA | NA | NA |
| Gandhi et al, 2021 [30] | NA (various people) | 700 videos^c^ | Various people | NA | CNN - VGG16:  60 ± 2%  CNN - VGG19:  65 ± 2%  Resnet50:  83 ± 2%  CNN + LSTM: 50 ± 5%  Resnet50 + LSTM: 50 ± 5%  OpenPose + LSTM: 97 ± 2% | NA | NA | NA | NA | NA |
| Uchil et al, 2019 [31] | 2 | 80 videos of 19 dynamic and 1 static sign for medical terms^c^ | 2 | Yes | 85% | NA | NA | NA | NA | NA |
| Areeb and Nadeem, 2021[23] | 26 | 2,060 frames from 412 videos: 60% were used for training, 20% for validation 20% for testing and 20% for validation | 26 | Yes (20% of the dataset) | 3D-CNN-based model: 82%  VGG16 combined with LSTM: 98%  You Only Look Once (YOLO) v5: 99.6% | Detection model: 99.6% | NA | NA | NA | NA |
| Adithya and Rajesh, 2020 [36] | 26 | 824 videos: 50% were used for training and 50% for testing | NA | Yes (50%of the dataset) | SVM: 90%  LSTM: 96.25% | NA | NA | NA | NA | NA |
| Ihsan et al, 2024 [37] | 20 | 3,596 videos: 80% were used for training and 20% for validation | NA | Yes (20% of dataset) | CNN BiLSTM Baseline (LSTM Unit = 256): 95.83% | CNN BiLSTM Baseline (LSTM Unit = 256): 93% | CNN BiLSTM Baseline (LSTM Unit = 256): 93% | CNN BiLSTM Baseline (LSTM Unit = 256): 93% | NA | NA |
| Das et al, 2024 [38] | NA | 288 videos, in which 75% were used for the training phase and 25% for the testing phase | NA | Yes (25% of dataset) | 67.53% | NA | NA | NA | NA | NA |
| Faisal et al, 2023 [39] | 33 signers | 145,035 videos: 58% used for training, 12% for validation and 30% for testing | 2 | No | 97.25% | NA | NA | NA | NA | NA |
| Bellil et al, 2024 [42] | NA | 100 videos (10 healthcare-related DZSL words, each recorded in 10 separate videos) | NA | NA | 100% | 100% | 100% | 100% | NA | NA |
| **Sensor-based (Depth-sensing)** | | | | | | | | | | |
| Hisham and Hamouda, 2019[29] | 5 | 1,260 samples: 840 were used for training and 420 for testing | 2 | No | NBC: 91.18% and RF: 92.5%  Accuracy enhanced after using Ada-Boosting: 93.7% | NA | NA | NA | NA | NA |
| Sarhan et al, 2015[33] | 4 | 215 instances (each signer performed each gesture at least  three times)^c^ | 4 | No (For signer independent experiment, the classifier was trained using all samples from three signers, leaving one signer  out for testing. This was repeated 4 times for each signer) | 80.47% | NA | NA | NA | NA | NA |
| Süzgün et al, 2015[35] | NA | 264 samples^c^ | 5 | No | 83% | NA | NA | NA | NA | NA |
| Dewasurendra et al, 2020 [41] | NA | NA | 8 (5 with mutism and 3 with hearing loss) | No | Test dataset  EfficientNet-Lite0 model: 99.76%  MobileNetV2 model: 96.16%  ResNet50 model: 88.71%  Unseen data  EfficientNet-Lite0 model: 68.21% | NA | NA | NA | NA | NA |
| **Sensor-based (Glove-based)** | | | | | | | | | | |
| Dere et al, 2022[32] | 2 | 10 videos: 5 were used for training and 5 for t testing | 2 | Yes (approximately 16,7% of the dataset) | 72% | NA | NA | 85% | NA | NA |
| Guo et al, 2023[34] | 6 | More than 550 words and 1,233 translations: 80% were used for training and 20% for testing | 12 | Yes (20% of the dataset) | Individual signs: 88.53%  Sentence Level:  91.37% | NA | NA | NA | NA | NA |
| Luqman and Mahmoud, 2020[40] | 2 | NA | 3 | No, three Arabic native speakers participated in the manual evaluation. The dataset was divided in training (70%), validation (15%) and testing (15%) | 92% (80% of the translations rated as understandable, 12% as somehow understandable, and 8% as not understandable) | NA | NA | NA | NA | NA |
| **Hybrid** | | | | | | | | | | |
| Sosa-Jiménez et al, 2022 [12] | 12 | 9,840 signs (each user performed each sign 10 times) in the training phase | 10,820 signs (each user performed each sign once) in the test phase | No |  |  |  |  |  |  |

|  | **User experience** |
| --- | --- |
| **Image-based** | |
| Xia et al, 2022 [13] | NA |
| Pikoulis et al, 2022 [14] | A source of errors in the proposed system is the extraction module, since the landmark-based features from hand tracking may not always give correct results in realistic conditions. |
| da Silva et al, 2024, [15] | Difficulty in classifying signals with similar phonemes.  Difficulty to recognize signs expressing pain without a component that acts directly on the signer’s face. |
| Das et al, 2023 S [27] | NA |
| Ko et al, 2019 [28] | The large visual variance between signers was one of the main difficulties encountered. |
| Barrientos-Villalta et al, 2022 [29] | NA |
| Ramirez Sanchez et al, 2021 [30] | NA |
| Gandhi et al, 2021 [32] | NA |
| Uchil et al, 2020 [33] | NA |
| Areeb and Nadeem, 2022[24] | The model achieved relatively better results in the signs "doctor" and "thief" which had the least movement. "Help", "lose" and "call" were not classified accurately. It could be that the model was not able to learn temporal features in the sequence. |
| Adithya and Rajesh, 2020 [38] | NA |
| Ihsan et al, 2024 [39] | NA |
| Das et al, 2023 [40] | NA |
| Faisa et al, 2023 [41] | NA |
| Bellil et al, 2024 [44] | NA |
| **Sensor-based (Depth-sensing)** | |
| Hisham and Hamouda, 2019 [31]^8^ | NA |
| Sarhan et al, 2015 [35] | NA |
| Süzgün et al, 2015 [37] | Five users tested the system and responded to a questionnaire, with 5 questions scaled from 1-5, in which the overall average response was 4.83 |
| Dewasurendra et al, 2020 [43] | A user satisfaction survey was conducted with 10 users.  User registration: high  Text-to-call: high  Call-to-sign: medium  Sign-to-call: medium  Pre-recorded message: high |
| **Sensor-based (Glove-based)** | |
| Dere et al, 2022[34] | NA |
| Guo et al, 2023[36] | A user study on signer’s experiences was conducted. Quality of Experience was rated on a 5-point scale: accessibility (4.2), usability (4.3), and overall experience (4.6). |
| Luqman and Mahmoud, 2019 [42] | Three Arabic native speakers evaluated each translation as Understandable (sentences that accurately preserve the meaning of the original Arabic sentence while also adhering to correct Arabic grammar and structure), Somehow Understandable (sentences that maintain the original meaning but contain grammatical or structural issues) and Not Understandable (sentences that convey a different meaning than the source sentence). |
| **Hybrid** | |
| Sosa-Jiménez et al, 2022 [12] | The recognition can fail if the arm position differs from one person to the other. Also, the performance of the Kinect may be affected by the sunlight and its use for several hours, because the device heats up. |

**^a^** Artificial Intelligence and Image Processing

^b^ Although the smart glasses include a gyroscope sensor, the system relies solely on image-based input (video) for sign recognition. Therefore, it is classified as an image-based system.

cDivision between the training phase and testing phase not reported.

CNN: Convolutional Neural Network; F1: F1-Score; HM: Hu Moments; Libras: Língua Brasileira de Sinais; LSM: *Lengua de señas mexicana;* LSTM: Long Short-Term Memory; NA: information not available; NBC: Naive Bayer Classifier; SL: sign language; SVM: Support Vector Machine; RF: Random Forest; TID: *Türk˙İşaret Dili;* ZM: Zernike Moments.
