# Supplementary material for: Innovations in Deaf Health Care Communication: Systematic Review of Sign Language Recognition Systems
Source: J Med Internet Res. 2026 Apr 9;28:e70417. doi: 10.2196/70417 (PMC13065231; doi:10.2196/70417)
Supplement: Multimedia Appendix 6 [file jmir-v28-e70417-s006.docx]

Technologies necessary for sign language recognition system’s implementation.

| **Categories** | **Subcategories** | **Image-based** | **Sensor-Based** | |
| --- | --- | --- | --- | --- |
|  |  |  | **Depth-Sensing** | **Glove-Based** |
|  |  |  |  |  |
| Hardware/Software | Processors (CPU) | Intel Core i3, Core i5, Kendryte K210 | Intel Core i7, Core i5 | NA |
|  | Operating systems | Windows7, MacOS Mojave, ROS, Ubuntu 18.04 LTS | Windows 7, Windows 8.1 | Android 10 |
|  | Image capture devices | Digital cameras, Mobile phones | Microsoft Kinect. Leap Motion Controller | NA |
|  | Data conditioning devices | NA | NA | Arduino Nano BLE |
|  | Sensors | NA | Microsoft Kinect | INMO AIR assistive AR glasses |
|  | Storage and cloud technologies | Google Cloud, Google Cloud Storage, Google Cloud Run | NA | NA |
|  | Softwares | Labelelmg, Autodesk Inventor software, Mobile Application User, Visual Studio Code, IClone Pro, 3DXchange, Unity | Microsoft Visual Studio 2015 | Edge Impulsive Software, |
| Development Technology (Artificial Intelligence and Image Processing) | Deep Learning Frameworks | TensorFlow, Keras | TensorFlow | NA |
|  | Image Processing Tools | Google Mediapipe, cuDNN 8.5 | Pillow (Python image processing library) | Android-MediaPipe |
|  | AI models and algorithms | YOLOv5, CNN, LSTM, SVM, RNN-LSTM, HMM, VGG-19, Sentence-BERT, Viterbi algorithm, Pre-trained CNN (MobileNetV2) model, model BiLSTM, LRCN model, Self-developed concise 3DGCN, TDNN model,  DTW, 3D CNN, InceptionV3 model, 1D-CNN model | Neural Networks, DTW, HMM, Bayesian Network, Ada-Boosting, Markov Models (probabilistic), EfficientNet-Lite0, MaryTTS, CMU Sphinx 4 toolkit, NLP techniques | 1D-CNN model, KenLM, MADAMIRA |
|  | Libraries | OpenPose Library, PyTorch, Kaldi toolkit, OpenCV library | NA | VOSK library |

3D-CNN: Three-Dimensional Convolutional Neural Network; 3DGCN: 3D Graph Convolutional Network; BERT: Bidirecional Encoder Representations from Transformers; BiLSTM: Bi-Directional LSTM; CNN: Convolutional Neural Network; DTW: Dynamic Time Wrapping; HMM: Hidden Markov model; LRCN: Long-Term Recurrent Convolutional Network; LSTM: Long Short-Term Memory; NA: not applicable; RNN: Recurrent Neural Network; ROS: Robot Operating System; SVM: Support Vector Machine; TDNN: Time Delay Neural Network; VGG: Visual Geometry Group; YOLO: You Only Look Once; NLP: Natural Language Processing; CMU: Carnegie Mellon University; KenLM: Statistical language model; MADAMIRA: Morphological analyzer and disambiguation tool; MaryTTS: Text-to-Speech framework
